# In Vitro and In Vivo Photoprotective Effects of (-)-Loliode Isolated from the Brown Seaweed, *Sargassum horneri*

**DOI:** 10.3390/molecules26226898

**Published:** 2021-11-16

**Authors:** Lei Wang, Hyun-Soo Kim, Jun-Geon Je, Xiaoting Fu, Caoxing Huang, Ginnae Ahn, Jae-Young Oh, K. K. Asanka Sanjeewa, Jiachao Xu, Xin Gao, In-Kyu Yeo, You-Jin Jeon

**Affiliations:** 1 College of Food Science and Engineering, Ocean University of China, Qingdao 266003, China; leiwang2021@ouc.edu.cn (L.W.); xiaotingfu@ouc.edu.cn (X.F.); xujia@ouc.edu.cn (J.X.); xingao@ouc.edu.cn (X.G.); 2 National Marine Biodiversity Institute of Korea, 75, Jangsan-ro 101gil, Janghang-eup, Seocheon 33662, Korea; gustn783@mabik.re.kr; 3 Department of Marine Life Sciences, Jeju National University, Jeju 63243, Korea; wpwnsrjs@naver.com; 4 Co-Innovation Center for Efficient Processing and Utilization of Forest Products, College of Chemical Engineering, Nanjing Forestry University, Nanjing 210037, China; hcx@njfu.edu.cn; 5 Department of Marine Bio Food Science, Chonnam National University, Yeosu 59626, Korea; gnahn@chonnam.ac.kr; 6 Food Safety and Processing Research Division, National Institute of Fisheries Science, Busan 46083, Korea; ojy0724@naver.com; 7 Department of Biosystem Technology, Faculty of Technology, University of Sri Jayewardenepura, Pitipana, Homagama 10206, Sri Lanka; asankasanjeewa@sjp.ac.lk; 8 Marine Science Institute, Jeju National University, Jeju 63333, Korea

**Keywords:** *Sargassum horneri*, (-)-Loliode, UVB irradiation, ROS, MMPs

## Abstract

Skin is the largest organ of humans. Overexposure to ultraviolet (UV) is the primary environmental factor that causes skin damage. The compound, (-)-loliode, isolated from the brown seaweed *Sargassum horneri*, showed strong antioxidant and anti-inflammatory activities in in vitro and in vivo models. To further explore the potential of (-)-loliode in cosmetics, in the present study, we investigated the photoprotective effect of (-)-loliode in vitro in skin cells and in vivo in zebrafish. The results indicated that (-)-loliode significantly reduced intracellular reactive oxygen species (ROS) level, improved cell viability, and suppressed apoptosis of UVB-irradiated human keratinocytes. In addition, (-)-loliode remarkably attenuated oxidative damage, improved collagen synthesis, and inhibited matrix metalloproteinases expression in UVB-irradiated human dermal fibroblasts. Furthermore, the in vivo test demonstrated that (-)-loliode effectively and dose-dependently suppressed UVB-induced zebrafish damage displayed in decreasing the levels of ROS, nitric oxide, lipid peroxidation, and cell death in UVB-irradiated zebrafish. These results indicate that (-)-loliode possesses strong photoprotective activities and suggest (-)-loliode may an ideal ingredient in the pharmaceutical and cosmeceutical industries.

## 1. Introduction

Skin is the largest organ and the first defensive line of the natural defensive system of the human body. Ultraviolet (UV) is the primary environmental factor that causes skin damage [1]. Based on the wavelength, UV can be classified into three subtypes, including UVA (320–400 nm), UVB (280–320 nm), and UVC (100–280 nm). UVB is characterized as causing more damage to human skin than UVA and UVC. This is because of its ability to penetrate the layers of the stratum corundum and epidermis [2]. UVB irradiation could cause skin damage such as thickening of the epidermis, pigmentation disorders, loss of elasticity, erythema, deep wrinkles, and skin cancer. Thus, resources that can be used to protect skin against UVB-induced photodamage have received the attention from researchers.

Natural products have the advantage of having high-effect and non- or low-toxicity. Thus, finding photoprotective materials from natural resources and developing a skincare agent to protect skin against the damage stimulated by UVB irradiation is an effective strategy for skin health. Numerous natural compounds have been reported to have photoprotective effect [3,4,5,6,7]. Zheng et al. investigated the photoprotective effects of theaflavins isolated from black tea. According to the findings, theaflavin-3′-gallate effectively protected human keratinocytes (HaCaT cells) against UVB-induced photodamage [6]. Li et al. evaluated the protective effect of the polysaccharide isolated from *Sophora japonica* L. flower against UVB-induced skin damage in HaCaT cells. The results suggest that the polysaccharide effectively decreased the UVB-induced apoptosis rate in HaCaT cells [7].

Seaweeds contain various bioactive compounds, such as polysaccharides, peptides, pigments, polyphenols, and sterols [8,9,10]. These bioactive compounds possess numerous health benefits, including antioxidant, anti-obesity, anti-cancer, anti-inflammatory, anti-melanogenesis, and UV protective effects [8,11,12,13]. Recent research indicates that algae-derived compounds possess strong photoprotective effects [11,13]. Fernando et al. have investigated the photoprotective effect of fucoidan (SSQC4) isolated from *Sargassum siliquastrum* [14]. The results indicated that SSQC4 effectively reduced intracellular ROS levels and apoptotic body formation, as well as improved the viability of UVB-irradiated HaCaT cells [14]. Ji et al. have reported the protective effect of polysaccharide from *Sargassum fusiforme* (SFP-P1) against UVB-induced oxidative stress in HaCaT cells [15]. The results indicated that SFP-P1 increased the activities of SOD and GSH-PX, and decreased the level of ROS [15].

**Figure 1:**
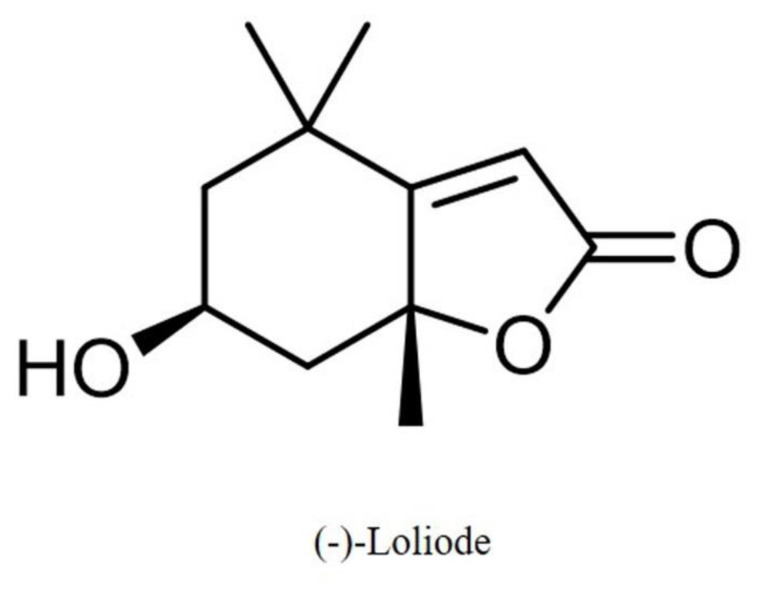
Structure of (-)-loliode.

In the previous study, a compound, (-)-loliode (Figure 1), has been isolated from *Sargassum horneri* and the antioxidant and anti-inflammatory activities of (-)-loliode had been investigated [16]. The results indicated that (-)-loliode possesses strong in vitro and in vivo antioxidant and anti-inflammatory activities and suggested the potential of (-)-loliode for photoprotective effects [16,17]. Therefore, in the present study, we investigated the photoprotective effect of (-)-loliode in vitro in human skin cells and in vivo in zebrafish.

## 2. Results and Discussion

### 2.1. Protective Effect of (-)-Loliode against UVB-Induced HaCaT Cell Damage

Skin is the largest organ in humans. It is directly exposed to environmental factors such as chemical and UV irradiation. UV irradiation is the primary environmental factor that causes skin damage. For the epidermis, UV irradiation could induce cellular damage such as apoptosis and necrosis by increasing the intracellular ROS level [18]. Thus, a compound with the ROS scavenging effect may possess the potential to protect skin against UVB-induced cellular damage. (-)-Loliode, an algae-derived compound, has been reported to possesses strong ROS scavenging effect in AAPH-stimulated Vero cells and zebrafish in our previous study [16]. In addition, Han, et al. reported that (-)-loliode suppressed oxidative stress and inflammation by activating Nrf2/HO-1 signaling in IFN-γ/TNF-α-stimulated HaCaT cells [19]. Furthermore, the methanol extract of *S. horneri* contains (-)-loliode showed photoprotective effect in vitro in HaCaT cells [20]. To further investigate the bioactivity of (-)-loliode and to explore its potential in the cosmeceutical industry, in the present study, we investigated the photoprotective effect of (-)-loliode in in vitro and in vivo models.

**Figure 2:**
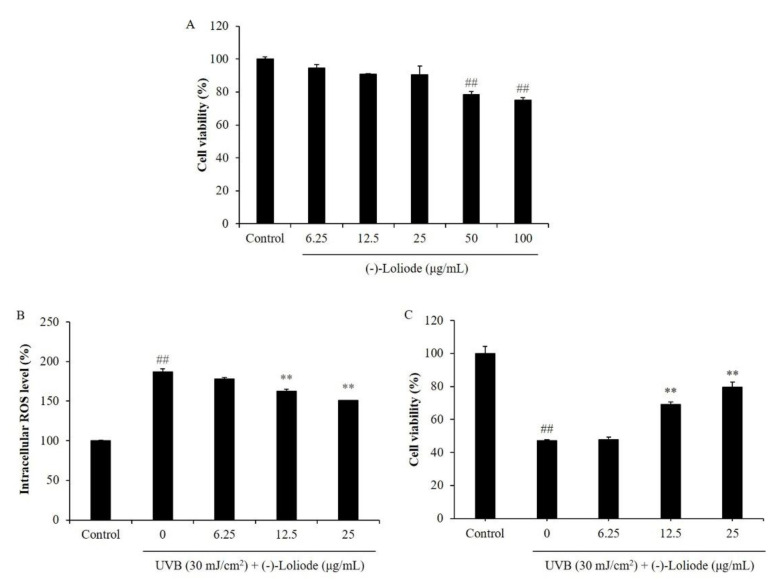
Protective effect of (-)-loliode against UVB-induced HaCaT cell damage. (**A**) Cytotoxicity of (-)-loliode on HaCaT cells; (**B**) intracellular ROS scavenging effect of (-)-loliode in UVB-irradiated HaCaT cells; (**C**) protective effect of (-)-loliode on UVB-induced cell death in HaCaT cells. Cell viability was measured by the MTT assay and intracellular ROS levels were measured by the DCF-DA assay. The data was expressed as the mean ± SE (n = 3). ** *p* < 0.01 as compared to the UVB-irradiated group and ^##^ *p* < 0.01 as compared to the control group.

Our results showed that (-)-loliode was showed cytotoxicity on HaCaT cells at the concentration higher than 50 μg/mL, but non-toxic at the concentration under 25 μg/mL (Figure 2A). Thus, the maximum concentration of (-)-loliode treat to HaCaT cells was determined as 25 μg/mL.

**Figure 3:**
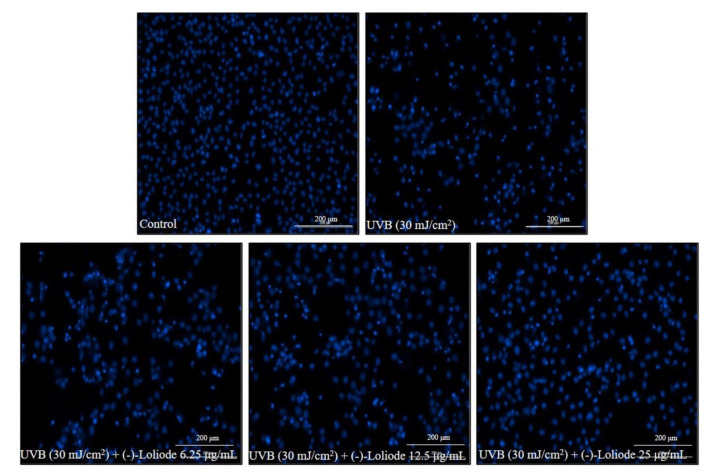
Protective effect of (-)-loliode against UVB-induced apoptosis in HaCaT cells. The apoptotic body formation was evaluated by Hoechst 33342 staining assay.

In the present study, the intracellular ROS level, the apoptotic body formation, as well as the viability of UVB-irradiated HaCaT cells were evaluated. As the results showed, UVB irradiation significantly increased the intracellular ROS level and decreased the viability of HaCaT cells; however, (-)-loliode remarkably and concentration-dependently reduced intracellular ROS level and improved the viability of UVB-irradiated HaCaT cells (Figure 2B,C). In addition, UVB irradiation significantly stimulated apoptotic body formation, and (-)-loliode effectively suppressed the apoptotic body formation in HaCaT cells (Figure 3). These results indicated that (-)-loliode effectively protected HaCaT cells against oxidative damage induced by UVB irradiation.

### 2.2. Protective Effect of (-)-Loliode against UVB-induced HDF Cell Damage

**Figure 4:**
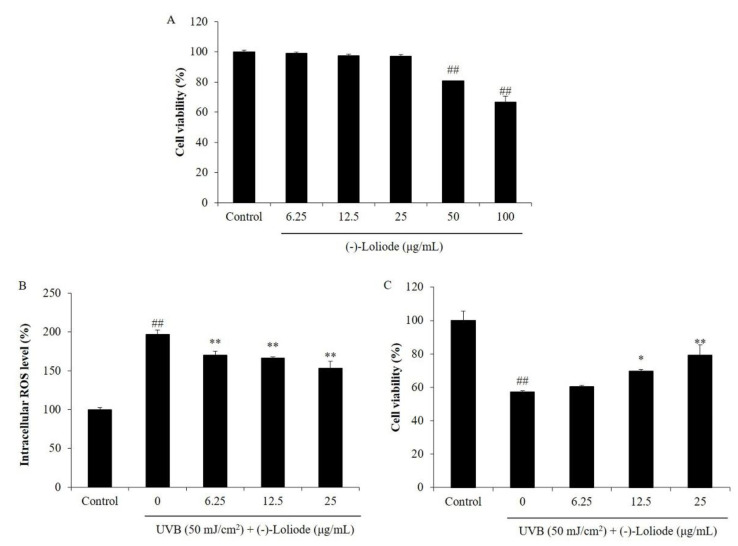
Protective effect of (-)-loliode against UVB-induced HDF cells damage. (**A**) Cytotoxicity of (-)-loliode on HDF cells; (**B**) intracellular ROS scavenging effect of (-)-loliode in UVB-irradiated HDF cells; (**C**) protective effect of (-)-loliode on UVB-induced cell death in HDF cells. Cell viability was measured by the MTT assay and intracellular ROS level was measured by the DCF-DA assay. The data were expressed as the mean ± SE (n = 3). * *p* < 0.05 and ** *p* < 0.01 as compared to the UVB-irradiated group, and ^##^ *p* < 0.01 as compared to the control group.

Previous studies indicated that UVB irradiation induces cell death, inhibited collagen synthesis, and increased MMPs expression by stimulating intracellular ROS generation in HDF cells [11,21]. Thus, in the present study, we investigated the effect of (-)-loliode on oxidative damage, collagen synthesis, and MMPs expression in UVB-irradiated HDF cells. According to the cytotoxicity analysis, (-)-loliode was non-toxic on HDF cells at the concentration under 25 μg/mL. Thus, the maximum concentration of (-)-loliode treatment on HDF cells was determined as 25 μg/mL. The intracellular ROS level of UVB-irradiated HDF cells was increased to 196.73% compared to the cells non-irradiated to UVB (100%) (Figure 4B). However, the intracellular ROS levels of the cells treated with 6.25, 12.5, and 25 μg/mL (-)-loliode were decreased to 170.21, 166.07, and 153.35%, respectively (Figure 4B). In addition, (-)-loliode increased the viability of UVB-irradiated HDF cells from 57.24% to 60.38, 69.72, and 79.31% at the concentrations of 6.25, 12.5, and 25 μg/mL, respectively (Figure 4C). The previous study has reported that the fucoidan isolated from *Hizikia fusiforme* decreased the intracellular ROS levels of UVB-irradiated HDF from 240.86% to 223.50, 208.67, and 203.14%, as well as improved the cell viabilities of UVB-irradiated HDF from 71.31 to 74.58, 78.34, and 81.17% at the concentration of 12.5, 25, and 50 μg/mL, respectively [21]. Compared to the present results, (-)-loliode showed a stronger photoprotective effect on HDF cells than the fucoidan isolated from *Hizikia fusiforme*.

**Figure 5:**
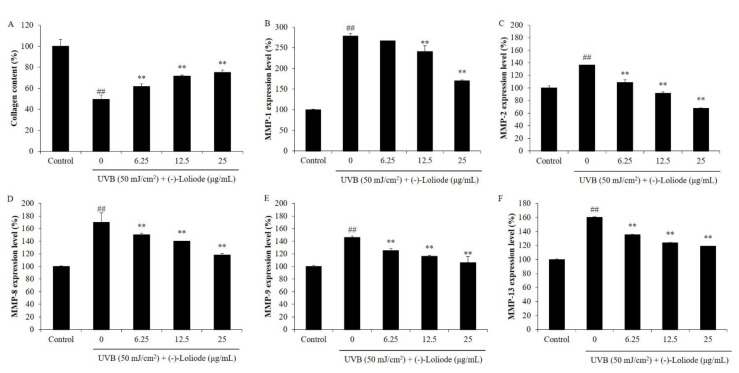
(-)-Loliode improves collagen content and inhibits the expression of MMPs in UVB-irradiated HDF cells. (**A**) Collagen contents in UVB-irradiated HDF cells; (**B**) MMP-1 expression levels in UVB-irradiated HDF cells; (**C**) MMP-2 expression levels in UVB-irradiated HDF cells; (**D**) MMP-8 expression levels in UVB-irradiated HDF cells; (**E**) MMP-9 expression levels in UVB-irradiated HDF cells; (**F**) MMP-13 expression levels in UVB-irradiated HDF cells. The amounts of collagen and MMPs were assessed using the ELISA kits following the manufacturer’s instructions. The data was expressed as the mean ± SE (n = 3). ** p < 0.01 as compared to the UVB-irradiated group and *^##^ p* < 0.01 as compared to the control group.

As Figure 5A shows, UVB significantly decreased the collagen level of HDF cells compared to the control group (100%). However, the collagen levels of the cells treated with (-)-loliode at the concentration of 6.25, 12.5, and 25 μg/mL were increased from 49.44% to 61.75, 71.72, and 75.78%, respectively (Figure 5A). Furthermore, UVB irradiation was significantly stimulated the expression of MMPs, particularly MMP-1 (Figure 5B–F). As the results showed, the MMP-1 level of HDF cells non-irradiated to UVB was thought as 100% and the MMP-1 level of UVB-irradiated HDF cells was increased to 278.64%. Whereas, (-)-loliode reduced the levels of MMP-1 to 266.58, 240.58, and 170.13% in UVB-irradiated HDF cells at the concentration of 6.25, 12.5, and 25 μg/mL, respectively (Figure 5B). These results demonstrated that (-)-loliode could effectively improve collagen synthesis and reduce MMPs expression in UVB-irradiated HDF cells.

In summary, the present results indicated that (-)-loliode protected UVB-induced photodamage in both epidermis and dermis cells. The protective effects were displayed in the following way: increased cell viability by inhibiting apoptosis via scavenging intracellular ROS in UVB-irradiated HaCaT cells; increased collagen content by improving oxidative damage and reducing MMPs expression in UVB-irradiated HDF cells.

### 2.3. Protective Effect of (-)-Loliode against UVB-induced Zebrafish Damage

Zebrafish have several advantages, such as the similarity of their genome to mammals, comparatively small size, and short life span. In recent decades, zebrafish have become a popular in vivo model in biological, toxicological, and pharmacological studies. UVB-irradiated zebrafish have been successfully used to investigate the photoprotective effect of natural compounds [1,21,22]. Previous studies indicated that UVB irradiation induces intracellular ROS generation, lipid peroxidation, nitric oxide (NO), and cell death in zebrafish, and these adverse effects could be suppressed by natural compounds [1,21,22]. Thus, in the present study, we evaluated the effect of (-)-loliode on UVB-irradiated zebrafish.

**Figure 6:**
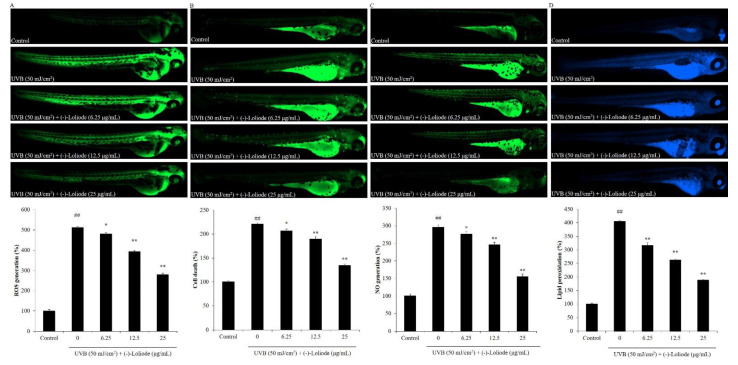
(-)-Loliode protects zebrafish against UVB-induced damage. (**A**) ROS generation of UVB-irradiated zebrafish; (**B**) cell death of UVB-irradiated zebrafish; (**C**) NO production of UVB-irradiated zebrafish; (**D**) lipid peroxidation of UVB-irradiated zebrafish. The relative fluorescence intensities of zebrafish were determined using Image J software. The data were expressed as the mean ± SE (n = 3). * p < 0.05 and ** p < 0.01 as compared to the UVB-irradiated group, and *^##^ p* < 0.01 as compared to the control group.

As shown in Figure 6A, UVB significantly increased the ROS levels of zebrafish, and (-)-loliode effectively and dose-dependently reduced ROS levels of zebrafish. In addition, (-)-loliode significantly suppressed cell death in UVB-irradiated zebrafish in a dose-dependent manner (Figure 6B). The NO generation of UVB-irradiated zebrafish was increased to 295.95% compared to non-irradiated zebrafish (100%). However, (-)-loliode decreased the NO levels to 276.63, 246.29, and 155.14% at the doses of 6.25, 12.5, and 25 μg/mL, respectively (Figure 6C). Furthermore, (-)-loliode suppressed lipid peroxidation stimulated by UVB irradiation in zebrafish in a dose-dependent manner (Figure 6D). These results indicated that (-)-loliode possesses a strong in vivo photoprotective effect in the zebrafish model.

## 3. Materials and Methods

### 3.1. Chemical and Regents

Dulbecco’s modified Eagle medium (DMEM), Ham’s nutrient mixtures medium (F-12 medium), trypsin-EDTA, penicillin-streptomycin (P/S), and fetal bovine serum (FBS) were purchased from Gibco-BRL (Grand Island, NY, USA). PIP ELISA kit was purchased from TaKaRa Bio Inc (Shiga, Japan). The 2,7-dichlorofluorescein diacetate (DCFH2-DA), 3-(4,5-Dimethylthiazol-2-yl)-2,5-diphenyltetrazolium bromide (MTT), 1,3-Bis (diphenylphosphino) propane (DPPP), diaminofluorophore 4-amino-5-methylamino-2′,7′-difluorofluorescein diacetate (DAF-FM-DA), and the ELISA kits used for the analysis of human MMPs were purchased from Sigma (St. Louis, MO, USA). All other chemicals used in this study were of analytical grade.

### 3.2. Preparation of (-)-Loliode from S. horneri

*S. horneri* was collected in June 2020 from the coastal area of Jeju Island, South Korea. (-)-Loliode was prepared according to the method described in our previous study [16]. In brief, the chloroform fraction from an 80% methanol extract of *S. horneri* was injected into high-performance centrifugal partition chromatography and separated using a solvent system composed of n-hexane/ethyl-acetate/methanol/distilled water (5:5:5:5, *v*/*v*). The isolated (-)-loliode was identified as a single compound using a high-performance liquid chromatography system and the structure of (-)-loliode was further confirmed by NMR spectra [16].

### 3.3. In Vitro in HaCaT cells

HaCaT cells (ATCC^®^ PCS-200-001™, Manassas, VA, USA) were cultured in DMEM medium (10% FBS and 1% P/S) and seeded at a density of 1 × 10^5^ cells/mL for experiments. To analyze the cytotoxicity of (-)-loliode on HaCaT cells, HaCaT cells were seeded and incubated with (-)-loliode (6.25, 12.5, 25, 50, and 100 μg/mL). After 24 h, the cell viabilities of (-)-loliode-treated HaCaT cells were measured by the MTT assay [23]. To evaluate the photoprotective effect of (-)-loliode in HaCaT cells, (-)-loliode-treated HaCaT cells were irradiated with UVB (30 mJ/cm^2^) in PBS solution (1×), then, the intracellular ROS level and the viability of UVB-irradiated HaCaT cells were investigated with the DCF-DA assay and the MTT assay, respectively [5]. In addition, the apoptosis body formation in UVB-irradiated HaCaT cells was detected with the Hoechst staining assay according to the protocol described by Wang et al. [24].

### 3.4. In Vitro in HDF cells

HDF cells (ATCC^®^PCS201012™, Manassas, VA, USA) were cultured in the medium mixed with F-12 and DMEM (1:3) supplemented with 10% FBS and 1% P/S. HDF cells were seeded at a concentration of 5.0 × 10^4^ cells/mL for experiments. To analyze the cytotoxicity of (-)-loliode on HDF cells, HDF cells were seeded and incubated with (-)-loliode (6.25, 12.5, 25, 50, and 100 μg/mL). After 24 h, the cell viabilities of (-)-loliode-treated HDF cells were measured by the MTT assay according to the method described by Wang et al. [23]. To evaluate the photoprotective effect of (-)-loliode in HDF cells, HDF cells were seeded and treated with (-)-loliode (6.25, 12.5, and 25 μg/mL). (-)-Loliode-treated cells were exposed to UVB (50 mJ/cm^2^) and the intracellular ROS level and the viability of UVB-irradiated HDF cells were determined with the DCF-DA assay and the MTT assay, respectively [11]. In addition, the collagen synthesis level and the expression of MMPs were assessed with ELISA kits (Sigma, St. Louis, MO, USA) using the cell culture medium [1,11].

### 3.5. In Vivo Assay

The zebrafish were maintained according to the conditions described previously [1]. The experiment was approved by the Animal Care and Use Committee of the Jeju National University (Approval No. 2019-O-0074). At 2 dpf, the zebrafish larvae (10 larvae/group) were treated with (-)-loliode (6.25, 12.5, and 25 μg/mL) for 1 h. The UVB-irradiated zebrafish larvae were further incubated for 6 h. The ROS levels, cell death, NO production, and lipid peroxidation of UVB-irradiated zebrafish were determined according to the methods described in our previous study [1].

### 3.6. Statistical Analysis

The experiments were performed in triplicates and the data are expressed as the mean ± standard error (SE). One-way ANOVA was used to compare the mean values of each treatment in SPSS 20.0. Significant differences between the means were identified with the Tukey’s test.

## 4. Conclusions

In this study, we investigated the photoprotective effect of the (-)-loliode isolated from *S. horneri* in vitro in HaCaT cells and HDF cells, as well as in vivo in zebrafish. The results indicated that (-)-loliode possesses strong in vitro and in vivo photoprotective effects, and suggested its potential in the cosmeceutical and pharmaceutical industries. To develop (-)-loliode as a therapeutic agent or cosmetic to treat and prevent UVB-induced skin damage, this clinical study is vital in further research.

## Data Availability

Not applicable.

## Sample Availability

Samples of the compounds are not available from the authors.
